# Piezoelectric Applications of Low-Dimensional Composites and Porous Materials

**DOI:** 10.3390/ma17040844

**Published:** 2024-02-09

**Authors:** Xiaoqiang Luo, Qingbin Li, Yichao Wang

**Affiliations:** 1 College of Chemical and Environmental Engineering, Pingdingshan University, Pingdingshan 467000, China; 2 School of Engineering, Design and Built Environment, Western Sydney University, Penrith, NSW 2751, Australia

**Keywords:** low-dimensional materials, piezoelectricity, composite materials, charge separation, technological applications

## Abstract

Low-dimensional (LD) materials, with atomically thin anisotropic structures, exhibit remarkable physical and chemical properties, prominently featuring piezoelectricity resulting from the absence of centrosymmetry. This characteristic has led to diverse applications, including sensors, actuators, and micro- and nanoelectromechanical systems. While piezoelectric effects are observed across zero-dimensional (0D), one-dimensional (1D), and two-dimensional (2D) LD materials, challenges such as effective charge separation and crystal structure imperfections limit their full potential. Addressing these issues requires innovative solutions, with the integration of LD materials with polymers, ceramics, metals, and other porous materials proving a key strategy to significantly enhance piezoelectric properties. This review comprehensively covers recent advances in synthesizing and characterizing piezoelectric composites based on LD materials and porous materials. The synergistic combination of LD materials with other substances, especially porous materials, demonstrates notable performance improvements, addressing inherent challenges. The review also explores future directions and challenges in developing these composite materials, highlighting potential applications across various technological domains.

## 1. Introduction

Piezoelectric materials have the ability to convert mechanical stimuli into electrical signals and vice versa, making them attractive candidates for use in a variety of applications, including sensors, actuators, and energy-harvesting devices [1,2,3,4,5]. The piezoelectric effect, first discovered in 1880, has been widely studied for its potential use in a range of technologies such as piezotronics, sensors, and energy-harvesting devices. Traditional piezoelectric materials, including ceramics and polymers, have been used for these applications due to their piezoelectric properties [6,7,8]. However, the limitations posed by the three-dimensional (3D) nature of these materials, particularly in micro- and nanoelectromechanical systems (MEMS/NEMS) due to size and stiffness constraints, have spurred a quest for innovative alternatives.

In recent years, there has been a growing interest in the development of low-dimensional (LD) materials, including 0D, 1D, and 2D materials, as potential candidates for piezoelectric applications due to their unique physical and chemical properties [9,10,11]. LD material-based piezoelectric materials have several advantages over traditional piezoelectric materials, such as piezoceramics. They are much more flexible and durable, which makes them suitable for use in a wide range of applications. They also have a higher piezoelectric coefficient, which means that they are able to generate more electrical energy from a given amount of mechanical strain. This makes them particularly suitable for use in energy-harvesting applications, where high efficiency is critical. LD materials have a high degree of flexibility, making them well suited for use in flexible devices such as soft robotics and wearable electronics. Additionally, due to their small size, LD materials can be easily integrated into miniaturized devices, making them attractive for use in micro- and nanoelectromechanical systems.

Exploring beyond traditional piezoelectric materials, the focus has now shifted to composite piezoelectric materials. These combine the strengths of traditional piezoelectric materials with unique properties from other materials, unlocking new performance levels and applications [12]. LD material-based composites can be created by combining LD materials with other materials, such as polymers, ceramics, or metals, to take advantage of the unique piezoelectric properties of both components. These composites have a range of potential applications, including sensors, actuators, and micro- and nanoelectromechanical systems. In recent years, there has been a significant amount of research focused on the development of LD material-based piezoelectric composites for use in energy harvesting and storage devices. These devices are designed to capture and store energy from ambient sources, such as vibration or temperature fluctuations, which can then be used to power small electronic devices or to charge batteries.

In addition to energy harvesting and storage, LD material-based piezoelectric composites have been explored for use in a range of other applications, including medical devices, automotive sensors, and structural health-monitoring systems. These applications often require materials that are able to withstand extreme environments, such as high temperatures, high pressures, or corrosivity. LD material-based piezoelectric composites are able to meet these requirements, making them an attractive choice for use in these applications.

Numerous review articles have extensively addressed the inherent characteristics of untouched piezoelectric materials, as well as exploring energy harvesting and flexible devices [4,13,14,15]. Nevertheless, there is a notable scarcity of systematic reviews on the piezoengineering of LD-based composite and porous materials. A more thorough review is essential to present the latest developments in this crucial area and underscore the prevailing trends.

This review provides a thorough overview of piezoelectric applications of low-dimensional composite and porous materials. Emphasis is placed on discussing how the structure of composite materials impacts the charge-separation, layer-dependent, and directional effects in low-dimensional materials. As a result, this review systematically summarizes the relationship between the material structure and application performance.

## 2. Fundamental Aspects and Evaluation Criteria of Piezoelectric Effects

### 2.1. Fundamentals and Methods for Characterization

When subjected to an external mechanical force or an electric field, piezoelectric materials can undergo polarization, leading to the generation of electric potential or displacement at a macroscopic level. The specified constants typically consist of two subscripts: the first one denotes the polarization direction within the piezoelectric material, while the second one indicates the direction of externally applied pressure or tension. In defining the polarization direction, subscripts 1, 2, or 3 are commonly substituted for the original X, Y, or Z directions, and the associated shear stresses are denoted by subscripts 4, 5, or 6 [3,16].

The performance indicators of piezoelectric materials are generally represented by the following parameters. Specifically, ‘d’ denotes the piezoelectric charge constant, measured in units of C/N or Pm/V (given that V/m = N/C). Meanwhile, ‘g’ signifies the piezoelectric voltage constant, with the unit Vm/N. Additionally, ‘ε’ represents the permittivity and ‘e’ corresponds to temperature-dependent factors, both measured in C/m^2^. ‘s’ means elastic compliance and the unit is m^2^/N. ‘K’ is the electromechanical coupling factor.

### 2.2. Piezoelectric Material Properties: Charge and Voltage Constants, Sensitivity, and Interconnections

In the study of piezoelectric materials, the piezoelectric charge constant, represented by ‘d’, holds significant importance. This constant signifies the polarization generated per unit under the application of mechanical stress (T) to the piezoelectric material or, conversely, the mechanical strain experienced by the material when a unit electric field is applied. External factors such as pressure or an electric field can induce polarization in piezoelectric materials. The piezoelectric charge constant, ‘d’, is characterized by two subscripts, where the first subscript denotes the direction of polarization within the material and the second indicates the direction of externally induced pressure or tension. For d_33_ and d_31_, both represent polarization in direction 3. In the case of d_33_, the applied pressure direction aligns with direction 3, whereas, for d_31_, the pressure is applied in direction 1 (perpendicular to the polarization direction) [17]. Another example is d_15_, where induced polarization occurs in direction 1, and the applied force is solely a shear force. The unit of measurement for the piezoelectric charge constant is [C/N], and its value is expressed in picometers per volt ([pm/V]).

Moving forward, the piezoelectric voltage constant, denoted by ‘g’, takes center stage. This constant defines the electric field generated by a unit force applied to a piezoelectric material or the electric displacement caused by applying an electric field on the material’s unit surface. Similar to the piezoelectric charge constant, ‘g’ also comprises two subscripts. The first subscript indicates the direction of the induced electric field or the generated electric displacement, while the second subscript signifies the direction in which the external force is applied.

The piezoelectric voltage constant, being a macroscopic representation of a material’s characteristics, serves as a key parameter for evaluating its suitability. Commonly encountered forms of ‘g’ include g_33_, g_31_, and g_15_. The first two, g_33_ and g_31_, indicate that the induced electric field aligns with directions 3 and 1, respectively, while the applied external force corresponds to these directions. On the other hand, g_15_ represents a horizontal induced electric field, with the applied external force being a tangential force in direction 5. The unit of measurement for the piezoelectric voltage constant is [Vm/N].

Proceeding to the magnitude of charges (sensitivity) concerning a given strain (e_ij_), the piezoelectric constant e_ij_ plays a crucial role. Its primary purpose is to eliminate the influence of mechanical clamping on the substrate, electrode, and surrounding unpoled film, ensuring accurate measurement using optical methods like a double-beam interferometer. The focus is often on e_31_ and e_33_, where e_31_ reflects the piezoelectric performance of the film and e_33_ represents the piezoelectric performance of the bulk material. Bulk piezoelectric materials typically exhibit two to three times the piezoelectric constant of the film. The piezoelectric constant e_ij_ essentially reflects the material’s response to changes in shape caused by external stress.

The piezoelectric constant (e_ij_ and d_ij_) is defined as the external strain (ε_j_) and stress (σ_j_) coefficients of the electric polarization change (P_i_, where i = x, y, z). The relationships between elastic constants, piezoelectric constants, and piezoelectric constants are articulated, emphasizing the interplay between these factors in response to external stress and electric field changes.

### 2.3. Methods for Measuring Piezoelectric Charge Constant

The integration of piezoelectric materials into actuators and sensors necessitates a thorough understanding of a crucial parameter—the piezoelectric charge constant. This constant, denoted as d_ij_, represents a material’s ability to generate charge under mechanical deformation and is measured in units of C/N or m/V [18,19]. Various methods are utilized to measure this coefficient, categorizing them into distinct groups. There are diverse methods for measuring piezoelectric charge constant offers and each has unique advantages and limitations, catering to specific experimental requirements and desired levels of precision in measuring the piezoelectric charge constant. Understanding these techniques is vital for accurate characterization and utilization of piezoelectric materials in diverse applications.

#### 2.3.1. Frequency Method Measurement

This approach involves the frequency method, which is particularly useful when a complete matrix of material coefficients is required. The accuracy of the resulting value depends on precise readings of resonance frequency and other necessary values. However, this method is limited by the requirement for first-order piezoelectric materials and specific sample constructions.

#### 2.3.2. Laser Interferometry Method (PFM)

Laser interferometry, a high-resolution technique, measures the displacement of a piezoelectric material’s surface under applied voltage [4,13,20]. Although sensitive, it is costly and susceptible to external vibrations. Laser interferometry is often used to measure specific piezoelectric charge coefficients (d_31_ and d_33_), with piezoresponse force microscopy (PFM) serving as a derivative device.

#### 2.3.3. Quasi-Static Method

The quasi-static method provides a cost-effective alternative, comparing piezoelectric samples with a known reference sample. This method measures both d_31_ and d_33_ coefficients without requiring complex processing. The charge on the sample is measured using a charge amplifier or voltmeter with high input resistance.

#### 2.3.4. MEMS Processing Technology (Berlincourt Method)

Microelectromechanical systems (MEMS) processing technology enables the generation of piezoelectric MEMS. The Berlincourt method utilizes a comb-drive actuator to produce dynamic forces and measures piezoelectric charges through charge-sensitive pre-amplifiers. This method is particularly effective for micro- and nanoscale measurements.

#### 2.3.5. Scanning Evanescent Microwave Microscope (SEMM)

SEMM is used to measure dielectric properties related to piezoelectrics and ferroelectrics. It relies on a high-quality microwave resonator and a sharp metal tip, demonstrating high sensitivity to tip-sample separation and enabling accurate measurement of tiny piezoelectric displacements [21].

#### 2.3.6. X-ray Diffraction Technique

X-ray diffraction is employed to observe polarization conversion, phase change, or structural deformation in piezoelectric materials. This method utilizes Bragg reflection and lattice deformation to measure piezoelectric effects. High-resolution synchrotron X-ray diffraction (HR-XRD) techniques provide exceptional resolutions [22].

#### 2.3.7. Laser Doppler Vibrometer (LDV)

LDV exploits inverse piezoelectric characteristics by applying a voltage of a certain frequency to tungsten electrodes on a piezoelectric material film. The resulting 180-degree phase shift is measured to determine the piezoelectric response.

## 3. Synthesis of LD-Nanostructures and Their Impact on Piezoelectric Properties

Fabrication methods for low-dimensional piezoelectric composites differ widely, depending on the desired dimensions and specific properties. Various approaches are employed to create these specialized materials with unique piezoelectric characteristics. Techniques such as sol–gel processes, chemical vapor deposition, and template-assisted methods are commonly utilized to engineer these composites at the nanoscale [23,24,25]. Additionally, bottom-up assembly processes, like layer-by-layer deposition and self-assembly [26,27], contribute to the controlled formation of low-dimensional structures. These fabrication methods play a crucial role in tailoring the mechanical, electrical, and piezoelectric performance of the composites, enabling their application in diverse fields, including sensors, energy harvesting, and biomedical devices.

The intricate world of piezoelectric materials, specifically those classified as LD materials, is predominantly governed by the atomic thickness film with a non-centrosymmetric structure. This structure, as detailed in prior research, is crucial for inducing piezoelectricity. Notably, the layered material must exhibit a specific charge delocalization, as metals lack piezoelectric properties due to shielding of polarization charges by a high electron concentration.

LD materials encompass zero-dimensional, one-dimensional, and two-dimensional entities. The challenge with 0D nanoparticles lies in their size, requiring a matrix as a carrier. Moving to 1D, nanowires, nanobelts, nanotubes, and nanorods take center stage, with diameters generally not exceeding 100 nm. Widely used materials in synthesizing piezoelectric nanorods include ZnO, GaN, and AlN with wurtzite structures. The realm of 2D introduces planar structures with nanometer thicknesses, and the recent trend involves the mixed use of 1D and 2D piezoelectric nanostructures.

Upon reviewing existing research, materials can be categorized into two types: layered materials, involving interlayer coupling, and single-layer films made of bulk piezoelectric materials. Layered piezoelectric films typically rely on weak van der Waals interlayers, featuring materials such as h-BN, TMDCs (2H-MoS_2_, 2H-WS_2_, 2H-MoSe_2_), and group III and IV monochalcogenides. Reduction to a single layer disrupts the centrosymmetrical structure, enhancing piezoelectric properties. Group III monochalcogenides like InSe, GaS, and GaSe display D3h point group symmetry, while Group IV monochalcogenides, including GeS, GeSe, SnS, and SnSe, exhibit a flexible folded C2V structure, demonstrating superior piezoelectric performance.

Furthermore, the directional influence on piezoelectricity in 2D materials becomes evident due to low-symmetry crystal structures, resulting in anisotropy. Taking black phosphorus (BP) as an example, its anisotropic characteristics showcase varying piezoelectric coefficients along different directions. Other materials, including rhenium disulfide (ReS_2_) and rhenium diselenide (ReSe_2_), also exhibit anisotropic piezoelectricity.

In the transition from bulk to a single layer, piezoelectric performance often improves, and materials with non-centrosymmetric 3D crystal classes present this opportunity. The lack of a center of symmetry in structures like wurtzite (ZnO, GaN, AlN, CdSe) and nanorods results in an asymmetric distribution of charges, leading to net polarization. ZnO, a representative piezoelectric material, demonstrates anisotropic properties due to its crystal structure. Additionally, the exploration of a single-phase organic–inorganic perovskite piezoelectric, trimethylchloromethyl ammonium trichloromanganese (II) (TMCMMnCl_3_), reveals remarkable piezoelectric coefficients under bulk conditions.

**Figure 1:**
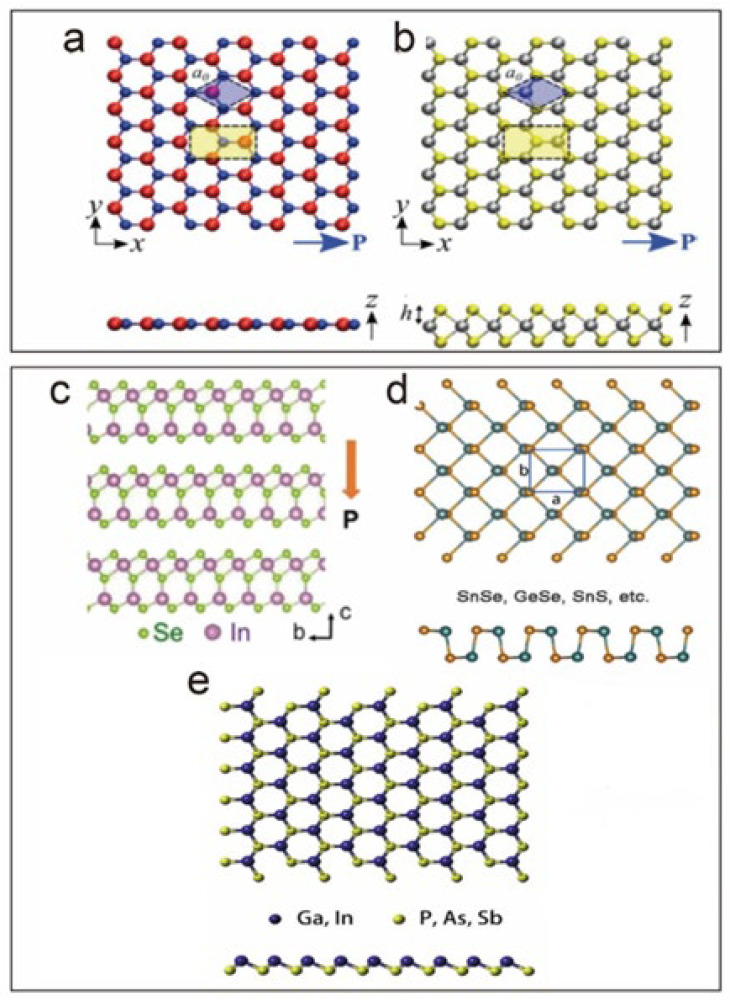
Structural model of two-dimensional piezoelectric materials. (**a**) Top view of atomically thin h-BN used for hexagonal and orthogonal unit cells. The DFT simulation is highlighted in blue and yellow. Reprinted with permission from ref. [28]. Copyright (2012) American Chemical Society, (**b**) Side view 2H-MoS_2_, which is representative of 2D layered piezoelectric materials of typical transition metal dichalcogenides. B, N, Mo, and S atoms are red, blue, silver, and yellow, respectively. The arrow indicates the direction of piezoelectric polarization. (**c**) Side-view crystal structure of In_2_Se_3_ as representative of group III compounds. The direction of piezoelectric polarization is indicated by the arrow (adapted from ref [29], copyright American Chemical Society, 2017) (**d**) Top view and side view of a typical group IV monosulfide monolayer. (**e**) The top and side views of the typical two-dimensional group III–V single-layer honeycomb [30].

Figure 1 provides a comprehensive structural model of various two-dimensional (2D) piezoelectric materials, showcasing a diversity of atomic arrangements and crystal structures. In panel a, the top view of atomically thin h-BN (hexagonal boron nitride) is depicted, illustrating hexagonal and orthogonal unit cells. The simulation using Density Functional Theory (DFT) is highlighted in blue and yellow, demonstrating the computational approach employed in understanding the material’s properties. For panel b, a side view of 2H-MoS_2_, a representative 2D layered piezoelectric material from the transition metal dichalcogenides family, is presented. The arrangement of atoms, including B, N, Mo, and S, is color-coded, and the arrow indicates the direction of piezoelectric polarization. Panel c introduces the side-view crystal structure of In_2_Se_3_, representing group III compounds, with the arrow indicating the direction of piezoelectric polarization. Panel d showcases the top and side views of a typical group IV monosulfide monolayer, offering insights into the atomic arrangement of this 2D material. Panels e and f present the top and side views of a typical two-dimensional group III–V single-layer honeycomb and an asymmetric moss monolayer structure, respectively. Finally, panel g reveals the structure of ZnO along the a-axis, highlighting the {000} and {0111} polar surfaces that are representative of the wurtzite structure. Collectively, Figure 1 serves as a visual guide to the diverse structures of 2D piezoelectric materials, providing a foundation for understanding their unique properties and potential applications in various technological domains.

## 4. Composite-Based Piezoelectric Materials

In the realm of enhancing piezoelectric properties, the technique of compositing low-dimensional materials with other compounds emerges as a powerful and efficient approach. This method capitalizes on the synergy of different components within the heterostructure, leading to elevated polarization levels and improved piezoelectric characteristics. The resultant heterostructures often exhibit superior energy-harvesting performance compared to individual components, owing to electrostatic fields and complex interfaces, resulting in nonzero charges and heightened moduli for enhanced energy-harvesting devices.

A significant aspect of this approach involves the conversion of nonpiezoelectric materials into piezoelectric ones through the formation of LD material-based composites. The combination of two entities in a composite induces piezoelectricity by disrupting the original centrosymmetric crystal structures. A notable example involves the enhancement of piezoelectric energy-harvesting properties in polarized poly (vinylidene fluoride–trifluoroethylene) [PVDF–TrFE] and graphene oxide (GO) bilayer films. The bilayer film demonstrates superior energy-harvesting performance compared to unipolar PVDF–TrFE films, showcasing the potential for diverse applications. Researchers such as Bhavanasi et al. and Rodrigues et al. have explored methods to induce piezoelectricity through compositing. Bhavanasi’s work involves enhancing the energy-harvesting properties of PVDF–TrFE and GO bilayer films, demonstrating improved performance with higher voltage and power output. Rodrigues, on the other hand, deposited a single-layer graphene onto SiO_2_ grating substrates, resulting in observable piezoelectric activities. These examples underscore the versatility of compositing LD materials for tailored piezoelectric applications. Mahmud et al. took a distinctive approach by integrating 1D and 2D ZnO nanostructures on a single substrate, deviating from traditional piezoelectric nanogenerators [31]. This innovative combination aimed to enhance the performance of piezoelectric nanogenerators by merging the advantages of 1D nanowires and 2D nanorods. In another study, Jae Cheol Shin reported that a 10% loading of nano-brick enhanced the remanent polarization from 0.04 to 6.10 μC cm^−2^ for PVDF, and the dielectric constant at 1 kHz increased by more than 5 and 6 times at 10% (ε_r_ = 13) and 20% (ε_r_ = 15.4) loading, respectively, compared to the pure PVDF matrix with an original dielectric constant of 2.5 [32].

The method of enhancing piezoelectric properties through compositing goes beyond engineering transformations. Instead, it focuses on combining different types of piezoelectric materials to amplify their collective properties. Heterostructures, particularly those involving PVDF, serve diverse applications, including actuators, energy storage devices, optoelectronics, and biomedical applications. The exploration of asymmetric layered structures within heterostructures facilitates the generation of electrostatic fields during deformation, contributing to the broadening scope of application scenarios. Therefore, LD materials and their composites present a promising avenue for advancing piezoelectric properties and diversifying applications. This approach holds substantial potential for shaping the future development of piezoelectric materials and their multifaceted applications.

## 5. Applications

Nanogenerators made of piezoelectric materials can effectively convert tiny kinetic energy into electrical energy, so they are widely used in medical monitoring sensors, environmental monitoring, wearable devices, energy generators, etc. Since, in 1880, the brothers Pierre Curie and Jacques Curie discovered the piezoelectric effect, many applications based on the piezoelectric effect have been created, especially the development of military technology experienced during the First World War and the Second World War. There have been many typical cases such as ultrasonic submarine detectors, echolocation devices, piezoelectric igniters, etc. The development of devices is also progressing with the development of new materials, as piezoelectric materials change from the original lithium niobate (LiNbO_3_), lead titanate (PbTiO_3_), quartz, and other materials to ceramics, lead-free piezoceramics, III–V and II–VI semiconductors, and polymers. The morphology of piezoelectric materials has gradually shifted from bulk form to 2D atomic-thickness film form. These innovations have promoted the tremendous development of devices. In recent decades, the main applications of piezoelectric materials are high voltage and power sources, actuators, frequency standards, piezoelectric motors, photovoltaics, and even the surgery field. In recent years, the research on piezoelectric materials has mainly focused on 2D. The synthesis of piezoelectric material films and the direction of ultra-thin materials have been the main directions of research on piezoelectric materials. And they also show huge advantages. The first is that the piezoelectric coefficient is higher than the bulk form. The second is that 2D material film has more electrical characteristics, and the third is that a 2D material provides the basis for the fabrication of flexible devices. The subsequent investigation summarizes the latest applications of 2D piezoelectric materials in recent years. The application of piezoelectric materials is mainly divided into two aspects: one is the mutual conversion of mechanical energy and electric energy, and the other is the production of photoelectric devices related to composites.

### 5.1. Mechanical or Strain Sensor

**Figure 2:**
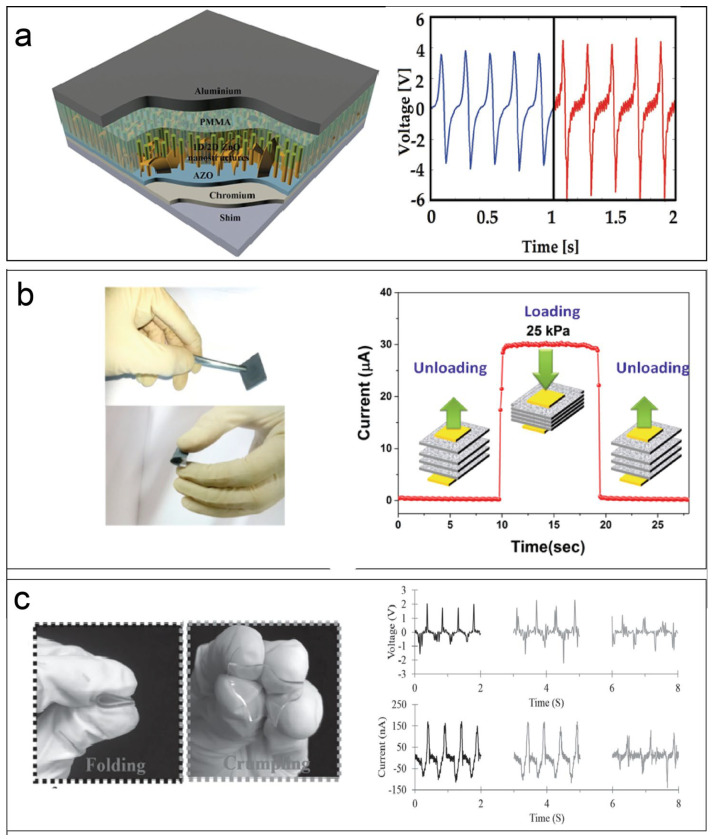
(**a**) 3D schematic of hybrid zinc oxide nanostructures and the output performance [31]. (**b**) The paper-based sensor fabricated by few-layer SnSe_2_ nanosheets and a single pulse of the time-resolved response with loading and unloading mechanism [34]. (**c**) Photographs of a graphene-piezoelectric fiber generator under different deformation types, and the corresponding output voltages and currents obtained [37].

**Figure 3:**
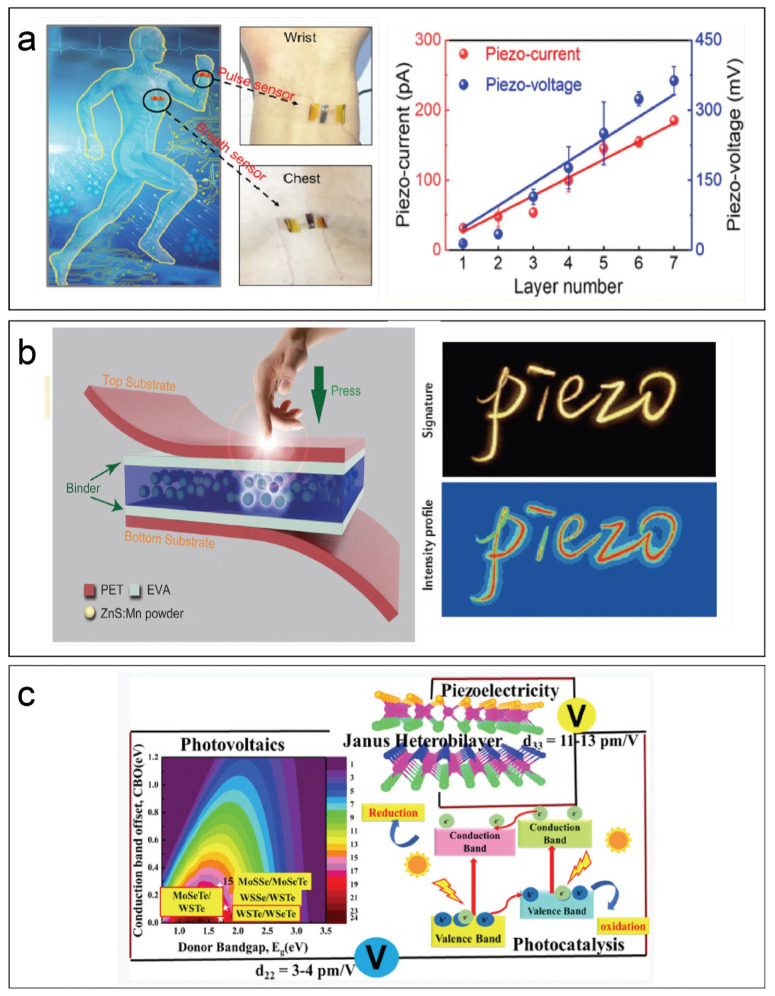
(**a**) Self-powered piezoelectric sensors can monitor physiological signals, pulse and respiration, when attached to the skin of the wrist (top right) and chest (bottom right), along with the piezoelectric current and voltage outputs [35]. (**b**) Pressure sensor matrix (PSM) device schematic configuration. The structure of the device with the characterization of ZnS:Mn particles. And visualization of the dynamic pressure distribution generated by writing “Piezo” by hand [38]. (**c**) The piezoelectricity, photocatalytic performance, and carrier mobility of Janus hybrid bilayers; the power conversion efficiency of 2D ultra-thin exciton solar cells composed of some heterobilayers is between 15 and 20%. The piezoelectric coefficient of the bilayer (d_33_ = 13.91 pm/V) is close to the piezoelectric coefficient of multilayer/bulk [39].

A mechanical or strain sensor piezoelectric device converts the surrounding mechanical stress or mechanical vibration due to mechanical stress into a piezoelectric response. On the contrary, it is possible to use an AC power source to make piezoelectric devices produce ideal vibrations. Examples of the mutual transformation of mechanical vibration or strain and piezoelectric response are mature, but, in recent years, as the thickness of piezoelectric materials tends to be at the atomic level, this has made flexible piezoelectric sensor fabrication possible. At present, the most novel sensor is a wireless wearable self-powered flexible electronic device. These wearable devices provide a wide range of applications for the development of intelligence and medical care, such as human health monitoring. Additionally, there are other application fields, for example, nanogenerators, resonators, sonar, texture energy harvesters, micromachined ultrasonic transducers, abd strain energy harvesters. Wearable devices generally require a flexible substrate. PVDF is used as a piezoelectric sub-material, and it is also a flexible polymer, which is an ideal substrate material. Mahmud et al. reported 1D/2D ZnO hybrid nanostructures grown on the same substrate (shown in Figure 2a). The output open-circuit voltage and short-circuit current had almost the same mechanical force in each cycle under the same measurement conditions, at a force of 5 N and a frequency of 5 Hz. The frequency average peak output open-circuit voltage and short-circuit current reach 10.18 V and 15.9 µA [31]. Mina et al. combined the piezoelectric properties of polyvinylidene fluoride (PVDF) nanofibers and the planner nanofillers graphene oxide and graphene; compared with PVDF powder, the electroactive phase (β-phase) of PVDF nanofiber felt increased by about 49% [33]. Mohit et al. dipped the 2D-SnSe_2_ nanosheet on Whatman filter paper; after frying and annealing, they produced packed SnSe_2_-decorated paper. They stacked this paper using Ag paste, and, finally, the sensor could monitor a human breath at rate of 3.2–3.5 s/breath (Figure 2b) [34]. Dai et al. reported real-time health monitoring by using seven-layer α-In_2_Se_3_; these flexible/wearable devices’ outputs could reach 0.363 V, with a current responsivity of 598.1 pA for 1% strain (Figure 3a) [35]. Zhang et al. demonstrated one ammonia (NH_3_) sensor driven by a novel flexible piezoelectric nanogenerator; the generator was fabricated using semiconductor MoS_2_ flakes [36].

There are many novel nanogenerators fabricated. As the earliest nanogenerator, Wang et al. used the two-dimensional odd number of layers of MoS_2_ to fabricate nanowire- and nanofilm-based nanogenerators [40]. Yiin et al., chose the piezoelectric polymer PVDF to fabricate piezoelectric fibers (Figure 2c) onto the surface of the monolayer and bilayer CVD-grown graphene. The graphene piezoelectric fiber generator (GPFG) showed highly transparent properties and achievable output voltage/current values of 2 V/200 nA [37]. Mengjun et al., explored a high-output flexible lead-free piezoelectric nanogenerator (PENG); they dropped inorganic piezoelectric 0.91K_0.48_Na_0.52_NbO_3_–0.04Bi_0.5_Na_0.5_ZrO_3_–0.05AgSbO_3_–0.2%Fe_2_O_3_ (KNN–BNZ–AS–Fe) particles on the PDMS matrix; this piezoelectric nanogenerator achieved an ultrahigh piezoelectric coefficient (d_33_) of 500 pC N-1 [41]. Gyoung-Ja et al. fabricated a piezoelectric energy harvester, using two-dimensional (2D) piezoelectric hexagonal boron nitride nanoflakes (h-BN NFs) deposited onto a flexible plastic substrate (polyimide, 125 μm). This harvester converted a piezoelectric voltage of ∼9 V, a current of ∼200 nA, and an effective output power of ∼0.3 μW [42]. In Figure 3b, Kar et al. reported one self-cleaning piezoelectric energy harvester; this harvester combined inorganic–organic 2D SnO_2_ nanosheet-/PVDF-based piezoelectric nanogenerators (PSNG). The output density could reach up to 4900 W·m^−3^ with an efficiency ca. 16.3% due to pressure gently imparted by a human finger [43]. Song et al., reported a piezoelectric nanogenerator/strain sensor; hexagonal PbI_2_ nanosheets were separated on the surface of polyethylene terephthalate substrate. The peak value of piezoelectric device open-circuit voltage, short-circuit current, and loading power were 29.4 mV, 20 pA, and 0.12 pW [44].

Nanogenerators are the main application method for converting mechanical energy into electrical energy. Furthermore, the transducers use the reverse mechanism; they are applied in many fields such as ultrasonic medical imaging, ultrasonic communication, ultrasonic range-finding, and handwriting input systems [45]. Park et al., reported honeycomb-shaped 1–3 connectivity piezoelectric micropillar arrays, with a crystal type having a size of 42 μm and an aspect ratio of 5 [46].

### 5.2. Photoelectric Sensor (No-Single-Mechanical or Strain Sensor)

In addition, there are non-single-mechanical piezoelectric sensors. This kind of sensor is generally heterogeneously combined with another characteristic material semiconductor material, so that the characteristics of the piezoelectric material, such as the high carrier concentration, can be used. Wi et al. developed a vertically stacked heterostructure photovoltaic device composed of indium tin oxide/n-MoS_2_/plasma-doped MoS_2_/Au, with a high short-ring photocurrent density of 20.9 mA/cm^2^ [47]. In Figure 3b, Wang et al. introduced one personalized handwriting application, wherein a flexible sensor records the habits associated with handwriting graphics and signatures. The realization of these features was based on the mechanical luminescence (ML) (ZMPs) of ZnS:Mn particles. Through the conversion process between mechanical stress and visible light emission, the single-point pressure and the two-dimensional planar distribution in the range of 0.6–50 MPa were instantaneously performed [38]. Rawat et al. formed a combined heterobilayer composed of two-sided monolayers, which was helpful to study the electronic, optical, piezoelectric, transmission, photocatalytic, and photovoltaic properties of “two-sided monolayers” relative to two-sided monolayers. Upgrading these heterobilayers can cause a large visible-light coefficient (α≈ 105 cm^−1^ (Figure 3c) [39]. Danget et al. designed one graphene field-effect transistor; the transistor mechanism was that the piezoelectric effect of ZnO NRs under static or dynamic pressure modulated the channel conductivity (σ) and caused a positive displacement of 0.25%/kPa at the Dirac point [48].

### 5.3. Thermal Applications

Thermoelectric materials use waste heat to generate electricity and will play an important role in global sustainable energy solutions. LD materials provide a new way for high-performance thermoelectric performance due to their unique electron-and-hole state density. As a typical representative of piezoelectric materials, MoS_2_ has very high Seebeck coefficients, on the order of ~10 mV/K, meaning that it is a representative material with the potential to make thermoelectric devices. Huang et al., used 1T phase MoS_2_ to show superior thermoelectric properties: the room temperature power factor could reach 73.1 μW m^−1^ K^−2^, which was much higher performance than the original graphene or single-walled carbon nanotubes [49]. Wang et al. reported a hybrid nanogenerator that included an electromagnetic generator (EMG), triboelectric nanogenerator (TENG), and thermoelectric generator, which collected both mechanical energy and thermal energy in one process [50]; thus, it was a hybridized nanogenerator for simultaneously scavenging mechanical and thermal energies by electromagnetic–triboelectric–thermoelectric effects [50]. In addition, Aly et al. tried to use a one-dimensional phononic crystal (PnC) that contained a piezoelectric material as a defect layer. Using the control pass band generated in the middle of the band gap, the position of the pass band within the band gap was adjusted by temperature changes. In this way, the application of an acoustic switch and a temperature sensor could be carried out [51] to determine thermal properties of a one-dimensional piezoelectric phononic crystal. Piezoelectric thermoelectric materials, as a new application of piezoelectric materials in recent years, have great potential as a new application field. There is a certain correlation between mechanical energy and thermal energy because thermoelectricity will also be accompanied by machinery in the future. This transformation will become more developed.

### 5.4. Biomedical Applications

**Figure 4:**
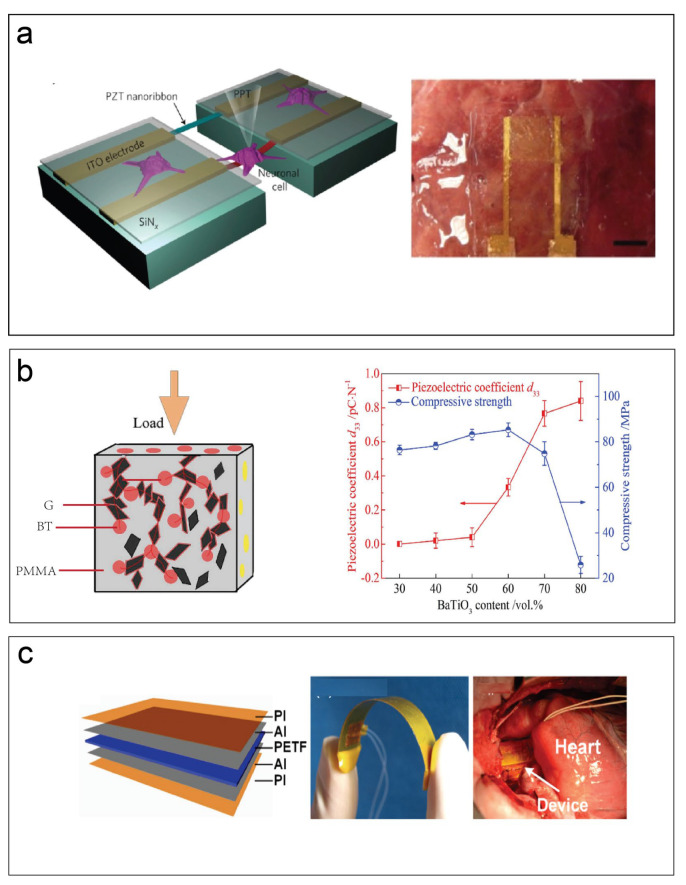
(**a**) Schematic diagram of the sensor device for suspending piezoelectric nanobelts and culturing PC12 cells. The mechanical deflection of the battery is caused by a glass pipette (PPT). PZT nanobelts can detect the deflection of cells and convert them into electrical signals. Then, the electrical signal is collected by indium tin oxide (ITO) electrodes, which are electrically isolated due to the SiN*_x_* coating [55]. Scale bar: 1 cm. (**b**) The mechanism of adding graphene to increase the piezoelectric coefficient of the G/BT/PMMA biopiezoelectric composite material, and the compressive strength of the BT/PMMA biopiezoelectric composite material’s piezoelectric coefficient d_33_ [56]. (**c**) The schematic diagram showing a device with a multilayer film structure. In turn, the photos of the device to the right show its flexibility (die > 1) and the device wrapped in a pig’s heart [57].

The development of piezoelectric materials in the medical field mainly depends on the great progress in microelectronics, such as the shrinking of electronic devices and the diversification of functions. The development of implantable medical products is mainly to improve the quality of human life, but also to help overcome human defects. Implantable medical electronics (IMEs) are implanted into the human body as diagnostic and therapeutic tools (in applications such as cardioverter defibrillators, sensors, cochlear implants, pacemakers, artificial retinas and stimulators (for nerves, brain, and bones), deep brain stimulation, piezoelectric acoustic sensors for biomimetic artificial hair cells, and tissue engineering). These implanted electronic devices provide diagnostic tools (such as monitoring of temperature, heart rate, and blood pressure) for several diseases in the body while supporting their treatment. At the same time, this can also help us to better understand the complex operating mechanisms in the human body, as seen in the developments in many biomedical fields in recent years [52]. Lee et al., studied the development of piezoelectric viruses, peptides, and other polymeric biomaterials. Their self-assembled thin film of phage exhibited piezoelectric strengths of up to 7.8 pm/V. Higher energy output could be obtained by arranging ordered 23 phage membranes in series or parallel [53]. Kapusetti et al., observed the piezoelectric scaffold placed on a predefined damage site, wherein the scaffold bore the functional load of the subject. The stent converted functional stress into electrical signals through piezoelectric phenomena. The generated synchronous electrical stimulation could regulate the Ca^2+^ channel, thereby enhancing the synthesis of various molecules and promoting the rapid regeneration of damaged bone tissue [54]. Electrical stimulation of cells and tissues is an important way to combine devices with living substances. Traditionally, standard stimulation methods are usually accompanied by destructiveness. Therefore, piezoelectric materials achieve indirect electrical stimulation. This possibility is a completely new solution for all biomedical research. As shown in Figure 4a, Marino et al., used piezoelectric nanomaterials to wirelessly deliver indirect electrical stimulation to neurons. By remotely activating the nanosensor, it was possible to induce Ca^2+^ and Na^+^ transients in neurons by activating the voltage membrane channel [55]. Tang et al., introduced one graphene/BT(BaTiO_3_)/PMMA biopiezoelectric composite. They incorporated BaTiO_3_ (BT) particles into biological materials, such as polymethyl methacrylate (PMMA) bone cement. The G/BT/PMMA biopiezoelectric composite material is not cytotoxic, and graphene can also promote cell adhesion and proliferation on the surface of the composite material (shown in Figure 4b). Polarized biopiezoelectric composite materials can improve cell morphology and promote cell proliferation [56]. Cheng et al., reported a blood pressure (BP) self-powered monitor with a piezoelectric thin film; the monitor had good linearity (R2¼ 0.971), the sensitivity was up to 14.32 mV/mmHg, and the maximum instantaneous power output in the body was 40 nW. This medical sensor integrated the coupling functions of energy harvesting and biomedical sensing and could effectively detect changes in cardiac blood flow (Figure 4c) [57]. The development of piezoelectric materials in the medical field is mainly based on the use of miniaturized generators as energy sources to perform various functional medical behaviors and on the stimulation of tissues by weak electricity caused by the interactions during tissue movement. As a brand-new solution to solve medical problems, piezoelectricity has huge development potential in the medical field.

Overall, the applications of piezoelectric materials can be divided into three categories. One, with the most numerous applications, is applications that convert the mechanical energy from vibration into electrical energy, such as many nano-generators, resonators, sonar, texture energy-harvesting wireless power supplies for wearable devices, etc. The second is the inverse application of the piezoelectric effect; the application is mainly electrical energy that is converted to mechanical energy, including resonators, ink injectors, piezoelectric motors, piezo-surgery, etc. The third is the heterogeneous combination of piezoelectric materials and other semiconductor properties. These possess the combination of piezoelectric properties and other electronic properties, and the two combined properties are used to make sensor devices, such as some transistors. However, in general, the future of piezoelectric devices will develop in the direction of flexible wearable devices. These are built based on flexible substrates, so PVDF is a key material. The development of this trend is also in line with the intelligent development of the Internet of Things.

## 6. Conclusions and Outlook

Piezoelectricity, a valuable phenomenon utilized in diverse applications such as sensing, thermal, biomedical, and energy-harvesting applications, is the focus of this review, which assesses various piezoelectric materials based on their properties, strengths/weaknesses, applications, and ongoing state-of-the-art research. The discussion delves into the materials science perspective of piezoelectricity mechanisms, categorizing materials into different composites, with an emphasis on the linear response in multiple directions. While ceramics traditionally exhibit superior piezoelectric coefficients and polymers offer flexibility and lightweight properties that are suitable for applications like wearable electronics, composite piezoelectric materials emerge as advantageous due to a well-balanced set of properties. However, challenges persist, notably in the efficiency of energy conversion and the need to match resonant frequencies with ambient vibrations for effective energy harvesting. Despite advancements, there is room for improvement in material properties, particularly in lowering resonant frequencies and addressing performance degradation at elevated temperatures. The review suggests avenues for enhancement, such as developing databases for existing composites to facilitate the creation of novel materials and the exploration of alterations in material architecture. Improved piezoelectric properties could lead to innovative applications, including self-powered ocean observation equipment and biocompatible nanocomposites powering cardiac pacemakers, ultimately positioning piezoelectric materials as crucial components in sensing and energy-harvesting technologies through composite optimization.
